# Circ-Spidr enhances axon regeneration after peripheral nerve injury

**DOI:** 10.1038/s41419-019-2027-x

**Published:** 2019-10-17

**Authors:** Susu Mao, Tao Huang, Yuanyuan Chen, Longxiang Shen, Shuoshuo Zhou, Shanshan Zhang, Bin Yu

**Affiliations:** 1 Key Laboratory of Neuroregeneration of Jiangsu and Ministry of Education, Co-innovation Center of Neuroregeneration, Nantong University, Nantong, 226001 China; 2 Department of Orthopedic Surgery, Shanghai Jiao Tong University affiliated Sixth People’s Hospital, Shanghai, 200233 China

**Keywords:** Molecular neuroscience, Peripheral nervous system, Regeneration and repair in the nervous system

## Abstract

Accumulating evidence suggests that circular RNAs (circRNAs) are abundant and play critical roles in the nervous system. However, their functions in axon regeneration after neuronal injury are unclear. Due to its robust regeneration capacity, peripheral nervous system is ideal for seeking the regulatory circRNAs in axon regeneration. In the present work, we obtained an expression profile of circRNAs in dorsal root ganglions (DRGs) after rat sciatic nerve crush injury by RNA sequencing (RNA-Seq) and found the expression level of circ-Spidr was obviously increased using quantitative real-time polymerase chain reaction (qRT-PCR). Furthermore, circ-Spidr was proved to be a circular RNA enriched in the cytoplasm of DRG neurons. Through in vitro and in vivo experiments, we determined that down-regulation of circ-Spidr could suppress axon regeneration of DRG neurons after sciatic nerve injury partially through modulating PI3K-Akt signaling pathway. Together, our results reveal a crucial role for circRNAs in regulating axon regeneration after neuronal injury which may further serve as a potential therapeutic avenue for neuronal injury repair.

## Introduction

Circular RNAs (circRNAs) have emerged as a large class of single-stranded regulatory RNAs in different species with complex tissue- and stage-specific expression patterns, which are predominantly produced from pre-mRNAs by back-splicing reactions^1,2^. CircRNAs have a higher degree of stability and sequence conservation among different species than linear RNAs and can be found in cell body, plasma, exosomes and cell-free saliva^3,4^, therefore they might be potential biomarkers or therapeutic targets. A large body of evidence indicates circRNAs are involved in various physiological and pathological processes^5^. At the molecular level, circRNAs are involved in gene expression by regulating transcription and splicing^6,7^, acting as miRNA sponges^8^, sequestering proteins to form specific circRNA-protein complexes that subsequently affect actions of associated proteins^2^, translating polypeptide^9^, and some circRNAs can act as sources of pseudogene derivation^10^. CircRNAs are preferentially derived from neural genes^11^ and highly abundant in the mammalian brain and neuronal cell lines^12,13^, indicating that they have potential neuronal functions. Furthermore, accumulating evidence indicates circRNAs contribute to various neurological disorders, such as pain^14^, Parkinson’s disease^15^, Alzheimer’s disease^16^ and neuronal autophagy^17^ and so on.

In the adult mammalian central nervous system (CNS), most of the axons are not able to regenerate after injury, while the injured neurons in the peripheral nervous system (PNS) have robust regenerative capability, which has been attributed to both inhibitory extrinsic signals and intrinsic growth ability of axons. Removing the extracellular axon regrowth inhibitors allows only a limited degree of axon regeneration in vivo^18^. Additionally, the axons of a unique kind of neurons, dorsal root ganglion (DRG) neurons, bifurcate into two separate branches: a central and a peripheral branch. A conditional lesion of the peripheral axon can increase the intrinsic growth ability of DRG neurons which is sufficient to enable the regeneration of their central axons in the CNS^19,20^. Hence, the intrinsic regeneration ability of neurons is believed to be primary in determining regenerative success. Previous studies have identified cytoplasmic signaling, transcriptional mechanisms and epigenetic mechanisms that promote intrinsic axon growth capacity^21–24^. However, the role of circRNAs in axon regeneration is largely unknown. Due to the amazing regenerative ability of peripheral nerve, sciatic nerve injury model is ideal for identifying the regulatory circRNAs in axon regeneration. In the present study, we employed sciatic nerve crush model to investigate the function of circRNAs and obtained an expression profile of circRNAs in DRG neurons after injury in rats by RNA-Seq. In addition, we determined the effect of circ-Spidr on axon regeneration of DRG neurons, which may provide new clues for studying the mechanisms underlying axon regeneration and present novel molecular targets for clinical therapy of nerve injury.

## Materials and methods

### Animal surgery and tissue preparation

All experimental procedures involving animals were performed in compliance with Institutional Animal Care guideline of Nantong University, and were ethically approved by the Administration Committee of Experimental Animals, Jiangsu Province, China. Thirty adult, female Sprague-Dawley (SD) rats (~200 g) were underwent surgery of sciatic nerve crush injury as previously described^25^. Briefly, the rats were anaesthetized by an intraperitoneal injection of complex narcotics (42 mg/kg magnesium sulfate (MilliporeSigma, Burlington, MA, USA), 85 mg/kg tri-chloroacetaldehyde monohydrate (MilliporeSigma) and 17 mg/kg sodium pentobarbital (MilliporeSigma), and the sciatic nerve was exposed by a small incision. The left sciatic nerve at 10 mm above the bifurcation into the tibial and common fibular nerves was crushed with a pair of forceps at a force of 54 N for 3 times (a period of 10 s for each time). The animals were then randomly divided into 6 groups, and the lumbar (L)4–5 DRGs were collected at 0 hour (h), 3 h, 9 h, 1 day (d), 4 d and 7 d after surgery respectively, and stored at −80 °C.

### Lumbar (L) 4/L5 intra-DRG injection

Female SD rats (~200 g) were randomly divided into two groups (*n* = 4 rats for each group). The intra-DRG injection procedure was described previously^25^. Briefly, the left L4 and L5 DRGs were exposed and the epineurium lying over the DRG was opened. For direct DRG injections, 2 n mol in 4 μl of 2OMe + 5Chol + 5Cy5 modified siRNAs (Ribobio, Guangzhou, China) were injected over a period of 5 minutes (min) into the L4/L5 DRGs through the indwelling catheter attached to a 10-μl Hamilton syringe. After a delay of 2 min, the needle was removed. After 48 h, the left sciatic nerve was crushed just as described above, and obtained for analysis another 72 h later.

### RNA extraction and RNA-Seq

Total RNA was extracted from the L4/L5 DRG tissues using TRIzol reagent (Thermo Fisher Scientific, Waltham, MA, USA) and then treated with amplification-grade DNase Ι (Thermo Fisher Scientific) according to the manufacturer’s instructions. For quantitation of circRNAs, RNase R (Epicentre, Madison, WI, USA) was used to degrade linear RNAs as described previously^26^. Total RNA from each sample was quantified by the NanoDrop 1000 spectrophotometer (NanoDrop Technologies, Wilmington, DE, USA). The total RNA samples (3 μg) were treated with the RiboMinus Eukaryote Kit (Qiagen, Valencia, CA, USA) to remove rRNA. And the cDNA libraries were generated according to the Illumina TruSeq RNA-Seq protocol and sequenced on an Illumina HiSeq 4000 sequencing platform. Data have been deposited under accession number SRP200823 in NCBI.

### Identification and annotation of circRNAs

For each RNA-Seq sample, FASTQ reads were first mapped to Rattus norvegicus reference genome (RGSC 5.0/rn5) obtained from UCSC genome database (http://genome.ucsc.edu/) by TopHat2^27^ and then CIRI2^28^ was used to identify circRNAs. The number of reads spanning back-spliced junctions was used as a measurement of circRNA abundance. While running CIRI2, default parameters were used for samples. All identified circRNAs were annotated according to the annotation of Rattus norvegicus reference genome. For each circRNA, we searched for transcript fragments that have overlap with the genomic position of circRNA and then defined the corresponding gene of this transcript fragment as the host gene of this circRNA. The circRNA donator/acceptor site was intersected to annotate gene regions, including exon, intron and intergenic regions.

### Quantitative real-time PCR (qRT-PCR) analysis

The purified total RNA was converted to cDNA using the PrimeScript RT Reagent Kit (TaKaRa Biotechnology Co. Ltd., Dalian, China) according to manufacturer’s protocol. Candidate circRNAs were characterized by PCR using the divergent primers annealing at the distal ends of the circRNAs given in Table S1 followed by agarose gel electrophoresis and Sanger sequencing. For quantitative RT-PCR, 5 ng cDNA were amplified in a 10 μl reaction containing SYBR Premix Ex Taq (TaKaRa) using a 2-step procedure. Melt curve analysis was enabled at the end of amplification. All samples were normalized using the 2^−ΔΔCT^ method against GAPDH and the experiment was repeated in triplicate. The primers used are listed in Table S2.

### Primary DRG neuron culture and transfection

Adult (8 weeks) rat DRGs were dissected in cold HBSS (Thermo Fisher Scientific) and then digested with 0.5 mg/ml collagenase (Roche, Basel, Switzerland) for 2 h at 37 °C followed by 0.125% trypsin (Thermo Fisher Scientific) digestion for 30 min at 37 °C. Tissues were triturated in culture medium (Neurobasal medium with 2% B27, 1% glutamine (Thermo fisher scientific)) with 1 ml tips and passed through a 70 μm cell strainer. Cells passing through the strainer were re-suspended in culture medium and plated to 24-well plates pre-coated with Poly-L-Lysine (MilliporeSigma). At the time of plating, neurons were transfected with siRNAs targeting circ-Spidr (circ-Spidr-si-1: CTGTAGAAATTTGCTAATG; circ-Spidr-si-2: GAAATTTGCTAATGGTGTC) (GenePharma, Shanghai, China) or lin-Spidr (lin-Spidr-si-1: CCAGGGCTCTTCAGTTTAA; lin-Spidr-si-2: CCCAGTGACAGTAGATGAA; lin-Spidr-si-3: GCTGTTGGAGAGCAGTATT) (GenePharma, Shanghai, China) using Lipofectamine RNAiMAX reverse transfection reagent (Thermo Fisher Scientific) according to the manufacturer’s instructions. After being cultured in transfection reagent overnight (12 h), neurons were transferred to fresh culture medium to allow incubation for additional 48 h. For in vitro axon regrowth analysis, cells were then re-suspended and re-plated to pre-coated cover slips. One day later, re-plated cells were fixed for Immunocytochemical analysis.

### Fluorescent in situ hybridization (FISH)

FISH was performed with a FISH Kit (RiboBio). In brief, primary cultured rat DRG neurons on coverslips were briefly rinsed in PBS and fixed with 4% paraformaldehyde (PFA) at room temperature (RT) for 15 min. Then the cells were rinsed in PBS for three times, 5 min for each time, and permeabilized in PBS containing 0.3% Triton X-100 at 4 °C for 5 min, washed with PBS three times for 5 min, and prehybridized at 37 °C for 30 min. Then the anti-circ-Spidr oligodeoxynucleotide probes (RiboBio) were added in the hybridization solution at 37 °C overnight in the dark. Cells were rinsed three times in 4× Saline Sodium Citrate buffer (SSC) for 5 min at 42 °C, followed by washing once for 5 min at 42 °C in 2× SSC and 1× SSC, respectively. Then the cells were counterstained with DAPI (Thermo Fisher Scientific) and imaged using a microscope.

### Immunocytochemical and immunohistochemical procedures

The primary cultured DRG neurons were fixed for 15 min in 4% PFA followed by 1 h blocking with 5% normal horse serum in PBS/0.3% triton X-100. Incubation with the primary antibody Tuj-1(ab18207, 1:1000, Abcam, Cambridge, MA, USA) was performed in 2% BSA overnight at 4 °C. For immunohistochemistry on tissue sections, rats were perfused transcardially with 100 ml PBS followed by 100 ml 4% PFA. The L4/L5 DRG tissues were removed, post-fixed in the same fixative overnight at 4 °C, and cryoprotected in 30% sucrose overnight. Cryostat sections (30 μm thick) were cut and processed for immunohistochemistry. The primary antibody rabbit polyclonal anti-SCG10 (NBP1–49461, 1:500, Novus) was used. Following the primary antibody, the secondary antibody Cy3-conjugated sheep anti-rabbit antibody (C2306, 1:1000, MilliporeSigma) was used. For quantitative analysis, SCG10 fluorescence intensity was measured along the length of the sciatic nerve using Image J and a regeneration index calculated by measuring the distance away from the crush site in which the average SCG10 fluorescence intensity is half that observed at the crush site^29^. The data were analyzed blindly by an investigator.

### FISH in combination with immunostaining

L4/L5 DRGs were cut into 20-μm sections which were fixed in 4% PFA at RT for 30 min and rinsed with DEPC-treated PBS twice and then incubated in a mixture (30% hydrogen peroxide: methyl alcohol = 1:50) at RT for 30 min, rinsed with DEPC-treated H_2_O for three times, and digested in 1 μg/ml Protease K at RT for 10 min. After permeabilization with 0.3% Triton X-100 in PBS for 15 min, the sections were prehybridized in hybridization buffer for 3 h at 40 °C. Hybridization buffer with 400 nM cy3-labeled circ-Spidr probe (RiboBio) was added dropwise to the sections, which were then hybridized at 40 °C overnight. The next day, the sections were rinsed three times in 4× SSC, twice in 2× SSC, and once in 1× SSC at 45 °C. The sections were blocked with 2% BSA in PBS for 1 h at RT and then incubated with mouse anti-NeuN antibody (MAB377, 1:500, MilliporeSigma) overnight at 4 °C. On the third day, after the samples were washed three times with PBS, they were incubated with Alexa Fluor 488 donkey anti-mouse IgG (A21202, 1:1000, Thermo Fisher Scientific) for 1 h at RT. The sections were washed three times in PBS. Finally, immunofluorescence images were captured using a microscope.

### Isolation of nuclear and cytoplasmic RNA

The nuclear and cytoplasmic fractions of primary DRG neurons were isolated and purified using the PARIS^TM^ Kit (Thermo Fisher Scientific) according to the manufacturer’s instructions. Total RNA from the nuclear or cytoplasmic fractions was isolated using TRIzol (Thermo Fisher Scientific). The relative expression level of circ-Spidr in each fraction was detected by qRT-PCR.

### Kyoto encyclopedia of genes and genomes (KEGG) pathway enrichment analysis

The list of the genes altered by knocking-down circ-Spidr was submitted to the database for annotation, visualization and integrated discovery (DAVID 6.8, https://david.ncifcrf.gov) for KEGG pathway analysis^30^.

### Western blot analysis

Seventy-two hours after transfection with NC-siRNA or circ-Spidr-si-2, the primary cultured DRG neurons were lysed using PIPA buffer (Thermo Fisher Scientific) and the proteins were extracted according to the manufacture’s instructions. Fifty micrograms of proteins were separated by 10% SDS-PAGE and transferred to PVDF membranes (Roche, Basel, Switzerland). The membranes were incubated in primary antibodies overnight at 4 °C. The following primary antibodies were used: rabbit monoclonal anti-AKT (ab179463, 1:2500, Abcam), rabbit monoclonal anti-p-AKT (phospho S473) (ab81283, 1:2500, Abcam), mouse monoclonal anti-β-actin (66009–1-lg, 1:2500, ProteinTech Group, Wuhan, China). Horseradish peroxidase affinipure goat anti-rabbit IgG (H + L) (111–035–003, 1:2500, Jackson ImmunoResearch, West Grove, PA) and horseradish peroxidase affinipure goat anti-mouse IgG (H + L) (115–035–003, 1:2500, Jackson ImmunoResearch) secondary antibodies were used. The signal was detected using the SuperSignal enhanced chemiluminescence system (Pierce, Rockford, IL, USA).

### Statistical analysis

The numbers of independent animals are described in the Materials and methods and Results sections or indicated in the figure legends. Data are expressed as mean ± SEM in bar and dot plots. Direct comparisons were made using two-tailed unpaired Student’s *t*-tests. Statistical significance was assigned, *P < 0.05, **P < 0.01. PRISM 5.0 software (GraphPad Inc., LaJolla, CA) was used for data analysis.

## Results

### Identification of circRNAs in DRGs after sciatic nerve injury

**Fig. 1:**
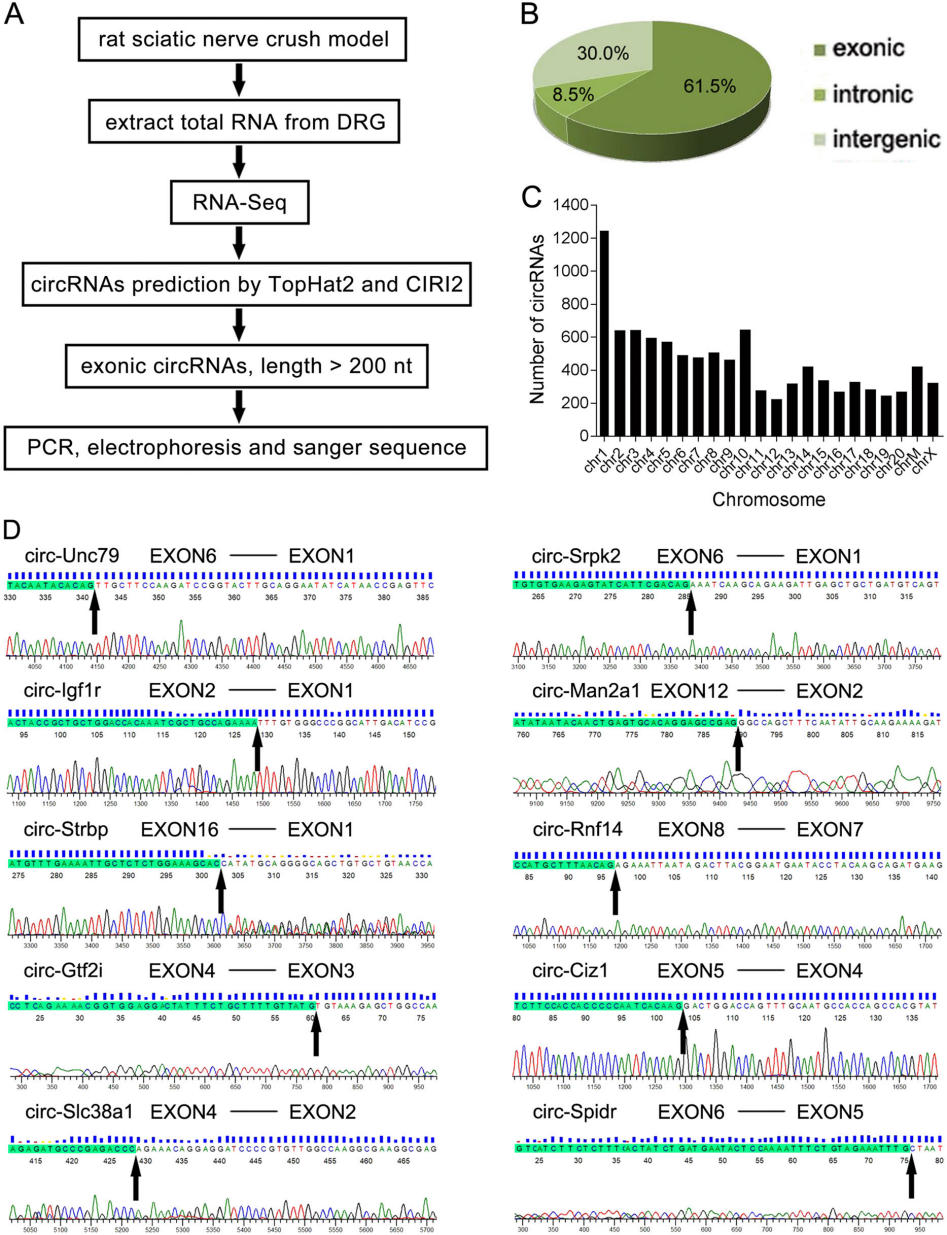
Identification of circRNAs in DRGs after sciatic nerve injury by RNA-Seq analysis. **a** Flowchart depicts work steps used to identify circRNA candidates of interest from RNA-Seq data. **b** Genomic origin of the circRNAs identified in rat DRGs. **c** Numbers of identified circRNAs in different chromosomes. **d** The 10 selected circRNAs were validated by Sanger sequencing. The black arrows indicate back-splicing sites

**Fig. 2:**
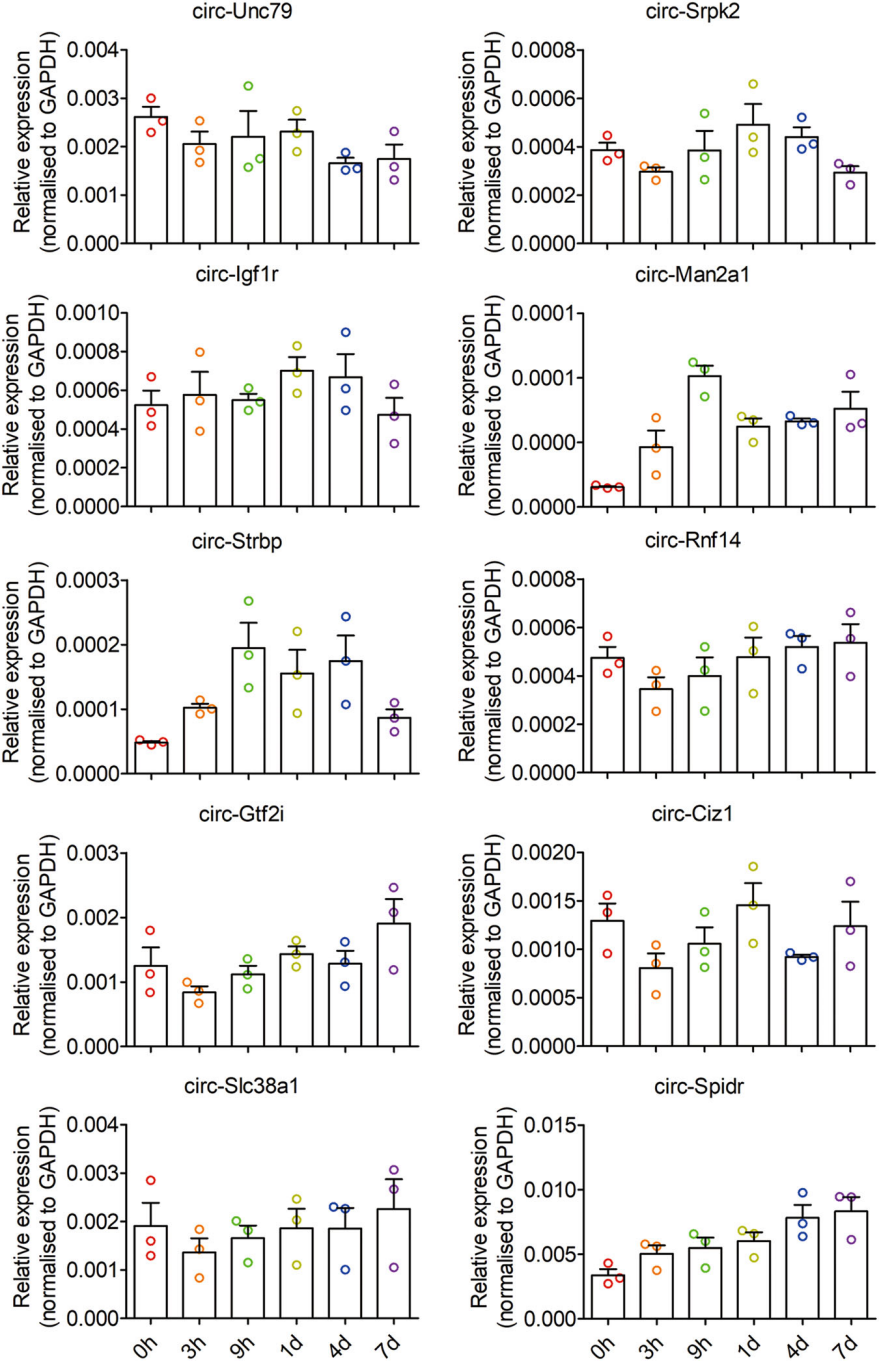
Expression changes of 10 candidate circRNAs in DRGs after sciatic nerve injury. The expression levels of 10 candidate circRNAs in DRGs at different time points post injury were analyzed by qRT-PCR. GAPDH was used as an internal control for normalization. Values are means ± SEM (*n* = 3 independent experiments)

In order to obtain an expression profiling of circRNAs during axon regeneration of DRG neurons after injury, we conducted a sciatic nerve crush injury model in rats. Then we isolated DRGs at various time points (0 h, 3 h, 9 h, 1 d, 4 d, and 7 d) post injury and performed RNA-Seq. Through bioinformatics analysis using Tophat2^27^ and CIRI2^28^, a total of 9821 circRNAs were identified (Fig. 1a, Table S3). A detailed description, including their junction reads, chromosome location, start and end sites, circRNA type, host gene id were shown in Table S3. According to their locations of the genome, these circRNAs were derived from exonic regions (61.5%), intergenic regions (30.0%), and intronic regions (8.5%) (Fig. 1b), indicating the circRNAs in DRGs are mainly derived from exonic regions, which is consistent with previous studies^31^. In addition, these circRNAs were widely and unevenly transcribed from all the rat chromosomes, including the mitochondrial genome, and the largest number of circRNAs were derived from Chromosome 1 (Fig. 1c). By the time series analysis with edgeR^32^, there were 1060 temporally differentially expressed circRNAs (FDR < 0.05, Table S4) in DRGs post injury. Based on the filter rule (RNA length > 200 nt and deriving from exonic regions), we randomly selected 12 circRNAs in Table S4 to validate their expressions by Sanger sequencing of PCR products, and 10 circRNAs were validated successfully (Fig. 1d). Then qRT-PCR was performed to analyze the expression changes of the 10 circRNAs in DRGs post sciatic nerve injury, which showed that circ-Man2a1, circ-Strbp, circ-Gtf2i, and circ-Spidr were obviously up-regulated, while circ-Unc79 was down-regulated (Fig. 2). Due to its highest expression level (0.0033 of GAPDH expression level), circ-Spidr was chosen for further functional study.

### Characterization of circ-Spidr

**Fig. 3:**
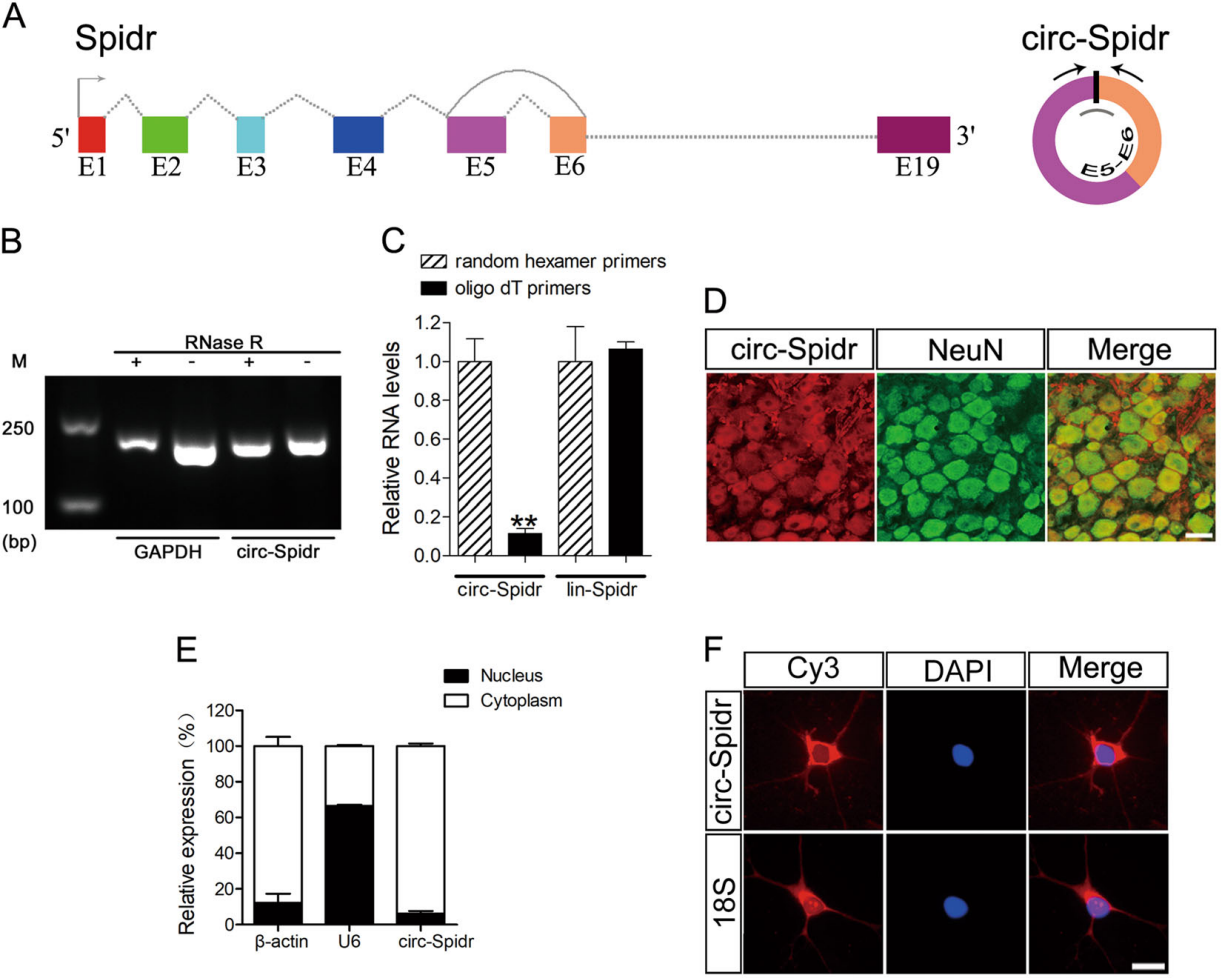
The characteristics of circ-Spidr. **a** The genomic locus of circ-Spidr in rat Spidr gene. In the right panel, arrows represent divergent primers binding to the genome region of circ-Spidr; the gray bar represents the siRNA against circ-Spidr; black bar represents the back-splicing site. **b** The cDNA derived from RNase R (+) or mock (−) treated total RNA in primary cultured DRG neurons was amplified with divergent primers for circ-Spidr. GAPDH was acted as a linear control. **c** Random hexamer or oligo (dT)_18_ primers were used in the reverse transcription experiments. The relative RNA levels of circ-Spidr and lin-Spidr were determined by qRT-PCR and normalized to the value using random hexamer primers. Values are means ± SEM. Asterisks indicate a statistically significant difference compared with the random hexamer primers of circ-Spidr group (**P < 0.01, unpaired two-tailed *t* test, *n* = 3 independent experiments). **d** Representative sections of a rat DRG. Red signals show the circ-Spidr + cells by FISH, while the green signals show the neurons by IHC. Scale bar = 50 μm. **e** Circ-Spidr is abundant in the cytoplasm of DRG neurons. β-actin and U6 were applied as positive controls in the cytoplasm and nucleus, respectively. Values are means ± SEM (*n* = 3 independent experiments). **f** RNA FISH analysis of circ-Spidr in cultured DRG neurons. Nuclei were stained with DAPI. 18S, probe for 18S rRNA, as a control. Scale bar = 10 μm

Circ-Spidr was derived from exons 5–6 of gene Spidr which has 19 exons (Fig. 3a). The RNase R digestion experiment showed circ-Spidr was resistant to RNase R (Fig. 3b) that can digest linear RNAs^33^. Compared with random hexamer primers, the detected level of circ-Spidr by qRT-PCR was significantly lower when the oligo (dT)_18_ primers were used, while that of lin-Spidr was not (Fig. 3c), indicating circ-Spidr had no poly-A tail indeed. These results confirmed the circular nature of circ-Spidr. The fluorescence in situ hybridization (FISH) in combination with immunostaining assay showed circ-Spidr was enriched in DRG neurons, even though its expression is not limited in neurons (Fig. 3d). In addition, qRT-PCR analysis of nuclear and cytoplasmic RNAs in primary cultured DRG neurons and FISH assay showed the predominant cytoplasmic distribution of circ-Spidr in DRG neurons (Fig. 3e, f). Collectively, these results demonstrate that circ-Spidr is a stable circRNA which is enriched in cytoplasm of DRG neurons, indicating it may have critical role in DRG neurons.

### Circ-Spidr promotes axon regeneration of DRG neurons in vitro and in vivo

**Fig. 4:**
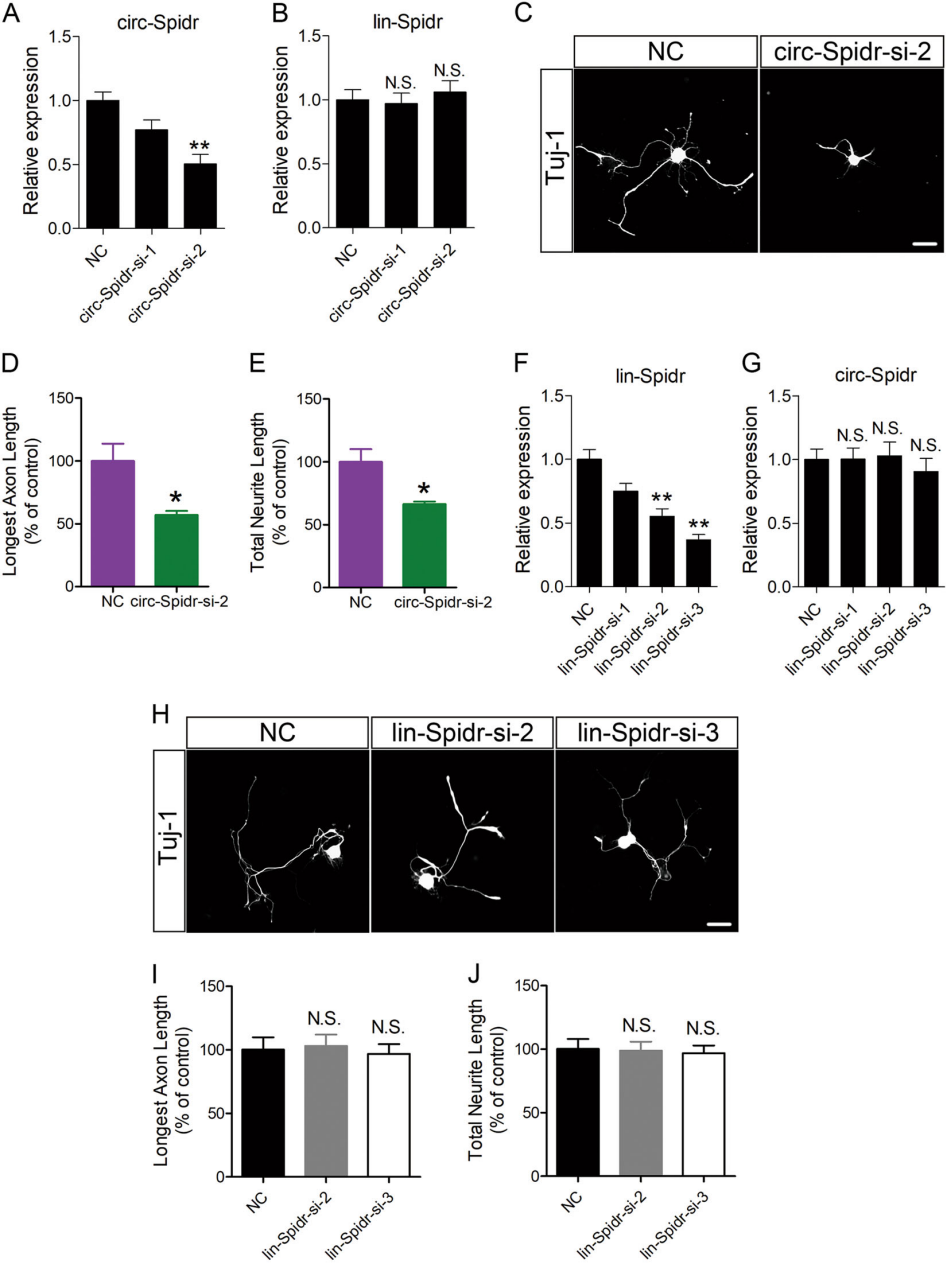
The function analysis of circ-Spidr on axon regrowth of DRG neurons in vitro. **a** The relative expression levels of circ-Spidr in DRG neurons transfected with its specific siRNAs or scramble siRNA (NC) were determined by qRT-PCR. Values are means ± SEM. Asterisks indicate a statistically significant difference compared with the NC group (**P < 0.01, unpaired two-tailed *t* test, *n* = 3 independent experiments). **b** The relative expression levels of linear Spidr (lin-Spidr) in DRG neurons transfected with siRNAs specifically against circ-Spidr were determined by qRT-PCR. Values are means ± SEM (N.S., not significant, compared with the NC group, unpaired two-tailed *t* test, *n* = 3 independent experiments). **c** Adult DRG neurons were dissected from rats and were treated with scramble siRNA (NC) or siRNA specifically against circ-Spidr (circ-Spidr-si-2); neurons were stained with Tuj-1. Scale bar = 20 μm. Longest axon length (**d**) and total neurite length (**e**) per DRG neuron were quantified. Values are means ± SEM. Asterisks indicate a statistically significant difference compared with the NC group (*P < 0.05, unpaired two-tailed *t* test, *n* = 3 independen*t* experiments). **f** The relative expression levels of lin-Spidr in DRG neurons transfected with its specific siRNAs or scramble siRNA were determined by qRT-PCR. Values are means ± SEM. Asterisks indicate a statistically significant difference compared with the NC group (**P < 0.01, unpaired two-tailed *t* test, *n* = 3 independent experiments). **g** The relative expression levels of circ-Spidr in DRG neurons transfected with siRNAs specifically against lin-Spidr were determined by qRT-PCR. Values are means ± SEM (N.S., not significant, compared with the NC group, unpaired two-tailed *t* test, *n* = 3 independent experiments). **h** Adult DRG neurons were dissected from rats and were treated with Scramble siRNA (NC) or siRNA specifically against lin-Spidr (lin-Spidr-si-2 or lin-Spidr-si-3); neurons were stained with Tuj-1. Scale bar = 20 μm. Longest axon length (**i**) and total neurite length (**j**) per DRG neuron were quantified. Values are means ± SEM (N.S., not significant, compared with the NC group, unpaired two-tailed *t* test, *n* = 3 independent experiments)

To determine the role of circ-Spidr in axon regeneration, we designed 2 siRNAs and found circ-Spidr-si-2 specifically inhibited expression of circ-Spidr but not lin-Spidr (Fig. 4a, b). Compared with control siRNA, transfection of circ-Spidr-si-2 significantly reduced the longest axon lengths and total neurite lengths (Fig. 4c–e), suggesting down-regulating circ-Spidr inhibits axon regeneration in vitro. To test whether the liner mRNA of Spidr (lin-Spidr) has the similar role with circ-Spidr in modulating axon regeneration, we knocked down the expression of lin-Spidr by specific siRNAs, which could only reduce the level of lin-Spidr, while not decrease the level of circ-Spidr (Fig. 4f, g). Compared with control siRNA, neither lin-Spidr-si-2 nor lin-Spidr-si-3 has any effect on the longest axon lengths and total neurite lengths of DRG neurons (Fig. 4h–j). These results suggest that circ-Spidr, instead of lin-Spidr, could enhance axon regrowth of DRG neurons in vitro.

**Fig. 5:**
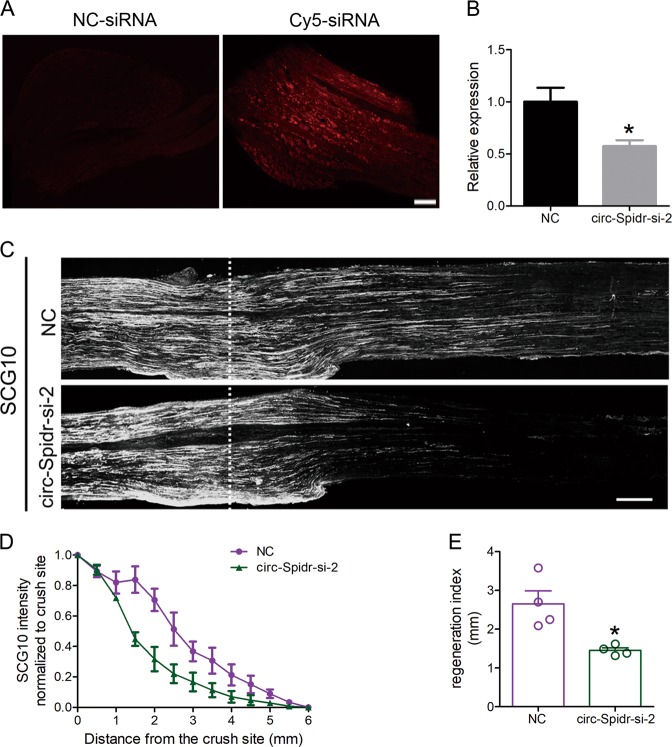
The function analysis of circ-Spidr on axon regeneration of DRG neurons after sciatic nerve injury. **a** Validation of in vivo siRNA injection by 2OMe + 5Chol + 5Cy5 modified siRNA. Three days after direct administration of siRNA, DRGs were harvested and photographed. The left panel showed the negative control siRNA-transfected (NC-siRNA) DRG; the right showed the 2OMe + 5Chol + 5Cy5 modified siRNA-transfected (Cy5-siRNA) DRG. Scale bar = 100 µm. **b** The relative expression levels of circ-Spidr in DRGs transfected with 2OMe + 5Chol + 5Cy5 modified specifically against circ-Spidr siRNAs (circ-Spidr-si-2) or 2OMe + 5Chol + 5Cy5 modified scramble siRNA (NC) in vivo were determined by qRT-PCR. Values are means ± SEM. Asterisks indicate a statistically significant difference compared with the NC group (*P < 0.05, unpaired two-tailed *t* test, *n* = 3 independent experiments). **c** Representative longitudinal sections from injured sciatic nerves. The crush site was indicated by white dotted line. Scale bar represents 500 µm. **d** Normalized SCG10 intensity plotted in function of the distance from crush line (*n* = 4 rats for each group). **e** Axon regeneration in injured rats is quantified by regeneration indices obtained from SCG10 immunostaining at 3 d after crush injury. Values are means ± SEM. Asterisks indicate a statistically significant difference compared with the NC group (*P < 0.05, unpaired two-tailed *t* test, *n* = 4 rats for each group)

To further substantiate down-regulation of circ-Spidr also could reduce axon regeneration capacity in vivo, 2′- O-methyl, 5′-cholesterol and 5′-Cy5 (2OMe + 5Chol + 5Cy5) modified circ-Spidr-si-2 was directly administered into L4–5 DRGs in rats, and significantly reduced the expression level of circ-Spidr in vivo (Fig. 5a, b). Two days after injection of modified circ-Spidr-si-2, the rats received a sciatic nerve crush injury and the nerves were dissected to label SCG10, a marker of regenerating sensory axons^34^ 3 days later. Circ-Spidr-si-2 treatment dramatically decreased the number of axons that regenerated past the crush site (Fig. 5c, d). Another index, a regeneration index, in which the average SCG10 signal intensity is half that observed at the crush site^35^, was employed to calculate the regeneration capacity. The regeneration index was significantly lower in nerves treated with circ-Spidr-si-2 compared with control siRNA-treated nerves (Fig. 5e). Collectively, these results indicate circ-Spidr has an ability to promote axon regeneration of DRG neurons after injury.

### Circ-Spidr functions by regulating PI3K-Akt signaling pathway

**Fig. 6:**
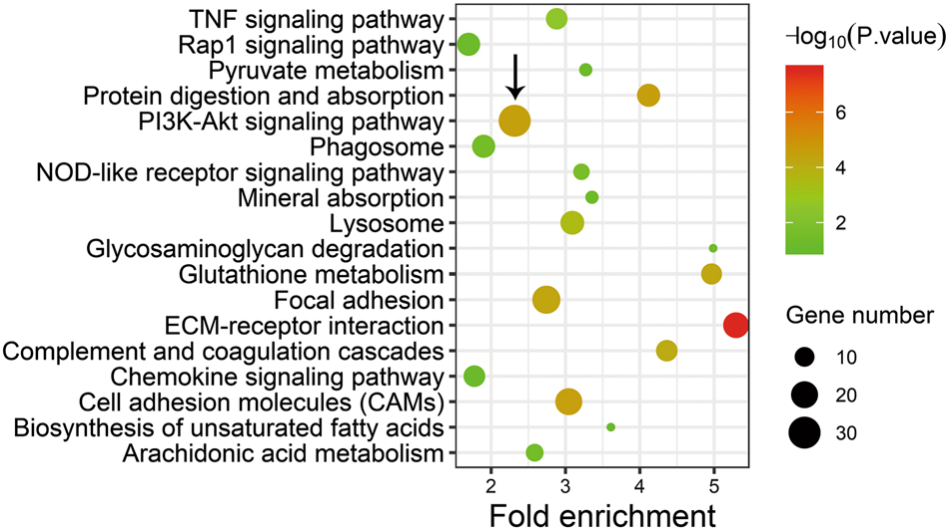
The KEGG pathway analysis of the genes altered by circ-Spidr. Scatter plot of enriched KEGG pathways statistics. KEGG pathway enrichments are displayed in a scatter diagram, where each point represents the enrichment level, the color corresponds to the −log_10_ (*P*-value), and the size corresponds to the number of genes enriched for the given pathway. Top 18 enriched pathways related to neuron injury repair are shown in the figure

**Fig. 7:**
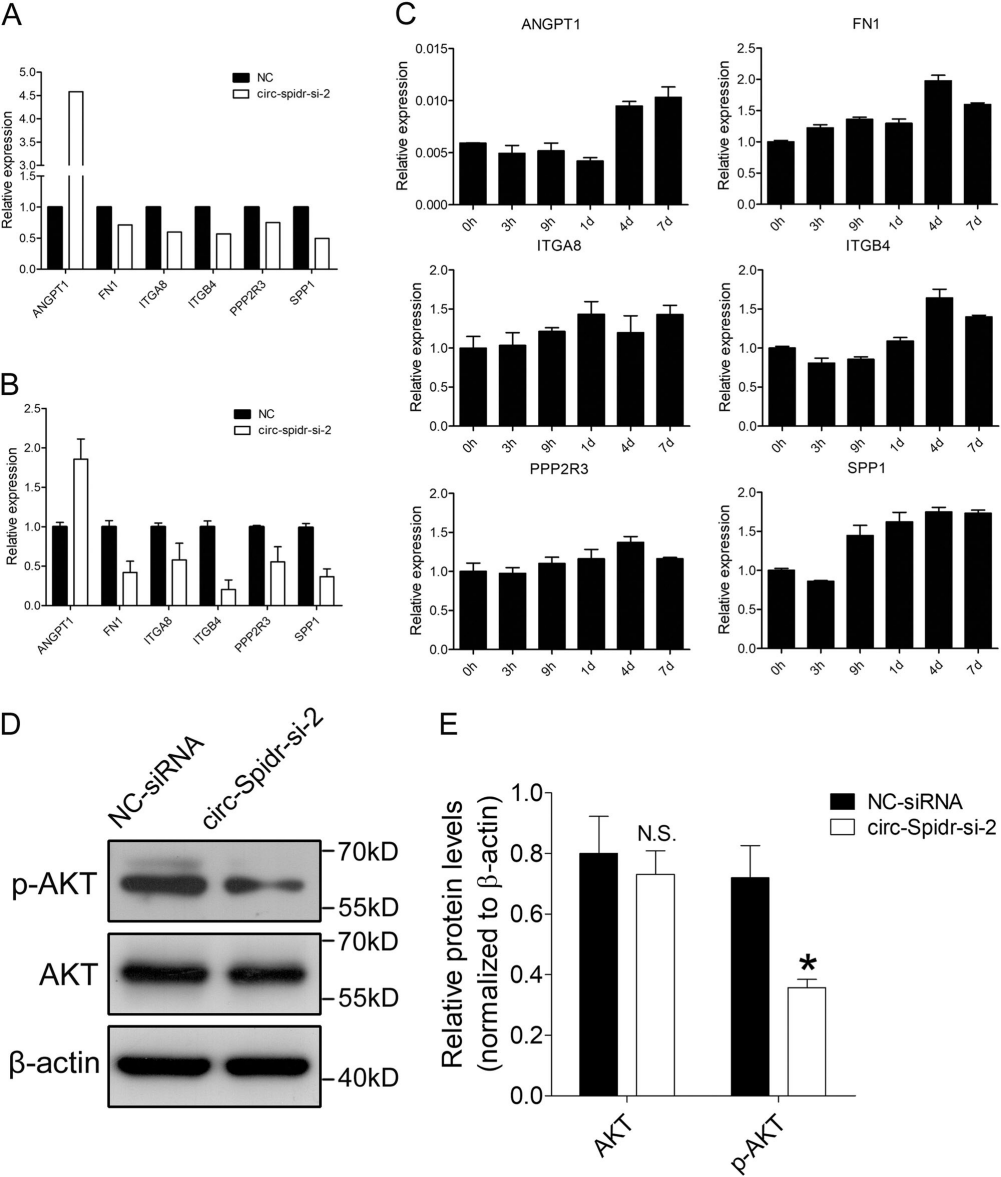
PI3K-Akt signaling pathway is modulated by circ-Spidr in DRG neurons. **a** The RNA-Seq data of the randomly selected six key genes enriched in PI3K-Akt signaling pathway in circ-Spidr-si-2 or the scramble siRNA transfected neurons were shown. **b** QRT-PCR validation of the six key genes in circ-Spidr-si-2 or the scramble siRNA transfected neurons. Values are means ± SEM (*n* = 3 independent experiments). **c** The expression levels of six selected genes in DRGs at different time points post injury were analyzed by qRT-PCR. Values are means ± SEM (*n* = 3 independent experiments). **d** Seventy-two hours after transfection with control siRNA (NC-siRNA) or siRNA against circ-Spidr (circ-Spidr-si-2), expression levels of AKT or p-AKT were analyzed by western blot. β-actin was used as an internal control. **e** Quantification of the protein expression levels from the experiment shown in (**d**). Values are means ± SEM (N.S., not significant, *P < 0.05, compared with the NC-siRNA group, unpaired two-tailed *t* test, *n* = 3 independent experiments)

To explore the mechanism underlying the effects of circ-Spidr on axon regeneration, we obtained the RNA from primary cultured DRG neurons transfected with circ-Spidr-si-2 or NC siRNA respectively, and performed RNA-Seq. Compared with NC siRNA, the circ-Spidr-si-2 significantly altered expression levels of 843 genes (*P* < 0.05, Table S5). For further analyzing the functions of circ-Spidr altered genes, KEGG pathway analysis was performed, which showed these differentially expressed genes were indeed involved in the pathways correlated with axon regeneration such as “PI3K-Akt signaling pathway”, “focal adhesion”, “cell adhesion molecules (CAMs)”, “protein digestion and absorption”, “complement and coagulation cascades”, “glutathione metabolism” and “ECM-receptor interaction”, among which the pathway with the largest number of genes enriched is “PI3K-Akt signaling pathway” (Fig. 6). Then we selected six genes to validate the RNA-Seq results (Fig. 7a) by qRT-PCR, and the assay results showed expression of ANGPT1 was increased, while that of FN1, ITGA8, ITGB4, PPP2R3 and SPP1 were decreased after down-regulation of circ-Spidr (Fig. 7b), which is consistent with the RNA-Seq results (Fig. 7a). Then we tested the expression changes of these genes in rat DRGs after sciatic nerve injury, and found all the six genes were increased after injury (Fig. 7c), which indicates the six genes have the similar expression trend with circ-Spidr (Fig. 2). However, given knocking down circ-Spidr upregulated the expression of ANGPT1 in vitro (Fig. 7a, b), while there was a similar expression trend between circ-Spidr and ANGPT1 in vivo, we could conclude that FN1, ITGA8, ITGB4, PPP2R3, and SPP1 might be the target genes of circ-Spidr except ANGPT1. To further verify the function of circ-Spidr on PI3K-Akt signaling pathway, we detected the AKT and p-AKT protein levels after knocking down the circ-Spidr in primary cultured DRG neurons. The western blot analysis showed protein levels of AKT were comparable between NC-siRNA and circ-Spidr-si-2 group, while the level of p-AKT was significantly reduced in the circ-Spidr-si-2 group compared with NC-siRNA group (Fig. 7d, e), which indicates down-regulation of circ-Spidr could inhibit the activation of PI3K-Akt signaling pathway in DRG neurons. Taken together, these data indicate circ-Spidr promotes axon regeneration at least partially through PI3K-Akt signaling pathway.

## Discussion

The non-reversible neurologic dysfunction in neurodegenerative diseases or after traumatic injury in CNS is mainly attributed to the failure of damaged axon to regenerate. Furthermore, lots of patients with peripheral nerve injury experience incomplete functional outcomes, possibly due to the slow rate of spontaneous axon regeneration. Thus, it is urgently needed to explore the strategies to enhance axon regeneration to improve functional recovery. Given the difference of axon regrowth ability between PNS and CNS, identifying how injured neurons in PNS switch to a pro-regeneration state will not only illuminate the biology process of axon regeneration in PNS, but also may provide potential novel therapeutic strategies for promoting axon regeneration in both PNS and CNS. Massive efforts have been made to explore the molecular mechanism underlying the axon regeneration of injured DRG neurons, which occurs mainly by down-regulating genes for neuronal activity along with neuronal maintenance, and up-regulating pro-growth transcriptional factors, such as ATF3, STAT3, Smad1, HIF-1α, c-Jun, and Sox11, as well as some growth-associated proteins such as GAP-43, SPRR1a, CAP-23 and so on^36–39^.

As a recent rising star, compared to other noncoding RNAs, such as microRNAs (miRNAs) and lncRNAs, circRNAs are more stable due to their specific tertiary structures and unique covalently closed loop, which offer more possibilities to act as novel therapeutic targets or ideal biomarkers. Recently, there is a growing body of evidence showing that circRNAs are involved in various physiological and pathological processes, such as development, cancers, and neurological disorders^40–42^. However, the role of circRNAs in axon regeneration remains unknown. In the present study, we found a large number of circRNAs in DRGs are differentially expressed after sciatic nerve injury, which may participate in the axon regeneration of injured DRG neurons. Furthermore, a pro-regeneration circRNA, circ-Spidr, was identified, which can enhance the axon regrowth after nerve injury. Several studies have indicated circRNA may play an irreplaceable role in neuron injury. For example, mmu-circRNA-015947 is involved in oxygen-glucose deprivation/reoxygenation-induced neuron injury; the circRNA chr8_87, 859, 283–87, 904, 548 promotes neuro-inflammation through increasing the CXCR2 by sponging let-7a-5p, blocking the neurological restoration after traumatic brain injury^43,44^. Nevertheless, it is the first time to find a circRNA which is involved in axon regeneration of injured neurons.

In this work, 1060 temporally differentially expressed circRNAs in DRGs after sciatic nerve injury were identified, among which circ-Spidr was determined to be significantly increased. Circ-Spidr is derived from gene Spidr, which is most highly expressed in the ovary and is involved in DNA double-strand break repair via homologous recombination, and hence contributes to maintain genomic integrity^45,46^. However, the function of Spidr in neuron injury repair has not been explored yet. Circ-Spidr was derived from exons 5–6 of gene Spidr, while there are 19 exons in lin-Spidr (Fig. 3a). In addition, unlike lin-Spidr, circ-Spidr has a covalently closed loop structure without the 5′ cap and 3′ poly(A) tail^5^. Their different nucleotide sequences and structure characteristics indicate circ-Spidr and lin-Spidr may have different biological functions. In the present study, we found circ-Spidr had the ability to promote axon regeneration of DRG neurons, while knocking down linear mRNA of Spidr had no effect on axon regeneration, indicating circ-Spidr has a linear mRNA-independent function, which is consistent with the conclusion that there is an independent role for circular transcripts in previous studies^47^.

To identify the mechanism underlying the effects of circ-Spidr, we knocked it down in DRG neurons by specific siRNAs and then got 843 differentially regulated genes. To further explore the potential relevance of differentially expressed genes changed by knocking down circ-Spidr, KEGG pathway analysis was performed to predict the functions of these target genes. In the significantly enriched pathways, there are several signaling pathways related with axon outgrowth. Based on previous studies, PI3K-Akt signaling is activated after peripheral nerve injury and is required for sensory axon regrowth which is induced by a transcription factor Smad^48^. Also, successful axon growth can be regulated by integrin interaction with the ECM, transduced by focal adhesion proteins^49^. In our previous study, we have identified some alternative splicing events of cell adhesion molecules which are involved in axon regeneration of DRG neurons^25^. In this work, the genes regulated by circ-Spidr were highly enriched in the above signaling pathways, especially in PI3K-Akt pathways. In addition, the qRT-PCR results in Fig. 7c showed some genes related to PI3K-Akt signaling were increased after sciatic nerve injury, which is also a proof indicating PI3K-Akt signaling is activated in sciatic nerve regeneration. There are already some evidences indicating circRNAs can regulate PI3K-Akt signaling. For example, ciRS-7 overexpression might enhance PTEN/PI3K/AKT pathway through inhibition of miR-7^50^, and circ-Amotl1 facilitates AKT activation and nuclear translocation in doxorubicin-induced cardiomyopathy to play a cardioprotective role^51^. These observations indicate circ-Spidr can modulate PI3K-Akt pathway to participate in axon regeneration. Previous studies have identified several general mechanisms of circRNA functions. For example, circRNAs can function as miRNA sponges to protect target mRNA from miRNA-dependent degradation; circRNAs may contain some protein binding motifs to regulate the protein function through directly circ-RNA-protein binding; a few circRNAs are shown to function as protein scaffolds to influence the reaction kinetics through inducing the colocalization of enzymes and their substrates; also, some circRNAs with internal ribosome entry site elements and AUG sites can be translated into peptides, and so on^52^. As circ-Spidr was mainly enriched in the cytoplasm, maybe, the possibility it can interact with transcriptional factors or other functional proteins in the nucleus can be eliminated. As regards the exact underlying mechanisms of circ-Spidr function, it needs further investigations.

Together, this study is the first to profile circRNAs expression in DRGs after sciatic nerve injury and demonstrate circ-Spidr can enhance axon regeneration of DRG neurons partially through regulating PI3K-Akt signaling pathway. Thus, circ-Spidr may be important as a target for therapeutic interventions in axon regeneration after nerve injury.

## Supplementary information

Table S1: Primers designed for PCR validation of candidate circRNAs

Table S2: Primers designed for qRT-PCR

Table S3: List of all identified circRNAs by RNA-Seq followed by bioinformatics analysis

Table S4: List of differentially expressed circRNAs (FDR<0.05)

Table S5: List of genes in primary DRG neurons transfected with circ-Spidr-si-2 or NC siRNA detected by RNA-Seq
