# Supplementary material for: Circ-Spidr enhances axon regeneration after peripheral nerve injury
Source: Cell Death Dis. 2019 Oct 17;10(11):787. doi: 10.1038/s41419-019-2027-x (PMC6797756; doi:10.1038/s41419-019-2027-x)
Supplement: Supplementary file 1 — Table S1: Primers designed for PCR validation of candidate circRNAs [file 41419_2019_2027_MOESM1_ESM.docx]

**Table S1: Primers designed for PCR validation of candidate circRNAs**

| NO. | circRNA | Host gene | Primers | Predicted Product length (bp) |
| --- | --- | --- | --- | --- |
| 1 | Circ-Unc79 | Unc79 | F: TACACGATGATCTCAACGCTAG  R: ACGGCACGGGATAAATG | 444 |
| 2 | Circ- Srpk2 | Srpk2 | F: TTTCCACCGTCTGGCTATG  R: GCCTTTCTGAAGAGGACGACT | 380 |
| 3 | Circ-Igf1r | Igf1r | F: ATGCGGTGTCCAATAACTACATT  R: TGTGAGGTTCGGGAAGAGG | 374 |
| 4 | Circ- Man2a1 | Man2a1 | F: GGAGGCAGATACGGTTGTCC  R: GCAAACGCTCCAAATGGTCG | 867 |
| 5 | Circ- Strbp | Strbp | F: TTGTTTGTTTCGTCCAGC  R: AAGGGTGTTGTGAATACGG | 494 |
| 6 | Circ-Rnf14 | Rnf14 | F: GGCTGCAACAAGATGACCTGT  R: ACCCTCTTGCCATACCTCTGT | 207 |
| 7 | Circ- Gtf2i | Gtf2i | F: TGCTTTTGTTATGTGTAAAGAGCTG  R: CGGTTCCAACGACAAACACA | 92 |
| 8 | Circ-Ciz1 | Ciz1 | F: CCAGGCTACTCGACAGTCCC  R: CATTGTAAGCACGGAGGTTGC | 226 |
| 9  10 | Circ- Slc38a1  Circ-Spidr | Slc38a1  Spidr | F: CCGTTAACTCGAGGCCACTT  R: TCCTCCTGTTTCTGGGTCTCG  F: ACCTGGTATTTGTGAGGCGA  R: AGAGGTCGACTAGCAGAACG | 141  207 |
