# Supplementary material for: Circ-Spidr enhances axon regeneration after peripheral nerve injury
Source: Cell Death Dis. 2019 Oct 17;10(11):787. doi: 10.1038/s41419-019-2027-x (PMC6797756; doi:10.1038/s41419-019-2027-x)
Supplement: Supplementary file 2 — Table S2: Primers designed for qRT-PCR [file 41419_2019_2027_MOESM2_ESM.docx]

**Table S2: Primers designed for qRT-PCR**

| NO. | | Gene name | Primers | Product length (bp) |
| --- | --- | --- | --- | --- |
| 1  2  3  4  5  6  7  8 | Spidr  ANGPT1  FN1  ITGA8  ITGB4  PPP2R3  SPP1  GAPDH | F: AAAAGGAAATCTGGCTGGTG  R: GTCCGTTCAATACAAACAATCTG  F: GCTGAACGGTTACACAGAGAG  R: GACGCTCTCCCCGTTAAAGA  F: CCCCAACTGGTTACCCTTCC  R: TGGTTCGCCTAAAGCCATGT  F: ATTCGGTAGTTCTGTGGCCC  R: CCGACAAGTAAATCTGGGTAATCA  F: AGTCCGAGGTAGTCCCACTC  R: CTTGTCCACTCGGATGCACT  F: GCAGCAGCTACTGTATAGTGAGAG  R: TCACAGTGCTAACCACGAGTC  F: CCAGCCAAGGACCAACTACA  R: TCTCCTCTGAGCTGCCAAAC  F: CCTTCATTGACCTCAACTACATG  R: CTTCTCCATGGTGGTGAAGAC | 211  185  199  258  176  233  111  215 |  |
